# On shape forming by contractile filaments in the surface of growing tissues

**DOI:** 10.1093/pnasnexus/pgac292

**Published:** 2022-12-12

**Authors:** Peter Fratzl, F Dieter Fischer, Gerald A Zickler, John W C Dunlop

**Affiliations:** Department of Biomaterials, Max Planck Institute of Colloids and Interfaces, Potsdam Science Park, 14476 Potsdam-Golm, Germany; Institute of Mechanics, Montanuniversität Leoben, 8700 Leoben, Austria; Institute of Mechanics, Montanuniversität Leoben, 8700 Leoben, Austria; Morphophysics Group, Department of the Chemistry and Physics of Materials, University of Salzburg, 5020 Salzburg, Austria

**Keywords:** tissue growth, mechanobiology, surface stress

## Abstract

Growing tissues are highly dynamic, and flow on sufficiently long timescales due to cell proliferation, migration, and tissue remodeling. As a consequence, growing tissues can often be approximated as viscous fluids. This means that the shape of microtissues growing in vitro is governed by their surface stress state, as in fluid droplets. Recent work showed that cells in the near-surface region of fibroblastic or osteoblastic microtissues contract with highly oriented actin filaments, thus making the surface properties highly anisotropic, in contrast to what is expected for an isotropic fluid. Here, we develop a model that includes mechanical anisotropy of the surface generated by contractile fibers and we show that mechanical equilibrium requires contractile filaments to follow geodesic lines on the surface. Constant pressure in the fluid forces these contractile filaments to be along geodesics with a constant normal curvature. We then take this into account to determine equilibrium shapes of rotationally symmetric bodies subjected to anisotropic surface stress states and derive a family of surfaces of revolution. A comparison with recently published shapes of microtissues shows that this theory accurately predicts both the surface shape and the direction of the actin filaments on the surface.

Significance Statement Growing tissues share many characteristics of fluids, for which the equilibrium shape is described by the classical Young–Laplace equation for isotropic surfaces. However, cells usually generate stress through contracting filaments, so that material behavior on the surface will not be isotropic. Here, we show that local mechanical equilibrium implies that contractile filaments should be along geodesics of the surface, and we completely characterize rotationally symmetric equilibrium shapes based on contractile fibers with constant contractile stress. Fibers are shown to typically wind in helical paths around the axis of rotation, in agreement with experimental observation.

## Introduction

Surface stresses have long been known to determine the shape of fluid bodies and they have been implicated in the growth of biological tissues. The general idea is that the minimization of the total surface energy leads to droplet shapes with constant mean curvature, whereby the associated surface stresses counterbalance a pressure inside the fluid droplet in a way described by the Young–Laplace equation [1, 2]. More than a hundred years ago, D’Arcy Thompson proposed that surface tension plays a role in the morphogenesis of biological organisms [3]. Indeed, growing tissues where cells permanently divide and rearrange behave—over sufficiently long timescales—as fluids [4, 5], where shear stresses relax over time and only isostatic pressure remains. D’Arcy Thompson’s ideas have meanwhile found their way into modern developmental biology, where the importance of mechanical forces for morphogenesis receives increasing attention [6, 7]. While a fluid droplet subjected only to surface energy would converge to the simplest shape with constant mean curvature, a sphere, the combination with the adherence to another wettable surface may lead to the emergence of complex shapes depending on the boundary conditions [8]. It has also been shown that surface energy influences not only the shape of growing tissues but also their growth kinetics [9], a phenomenon that can be rationalized through simple models of tissue growth [10]. Such concepts have significant practical implications for the optimal design of scaffolds for tissue engineering [11, 12], for example.

**Fig. 1.**
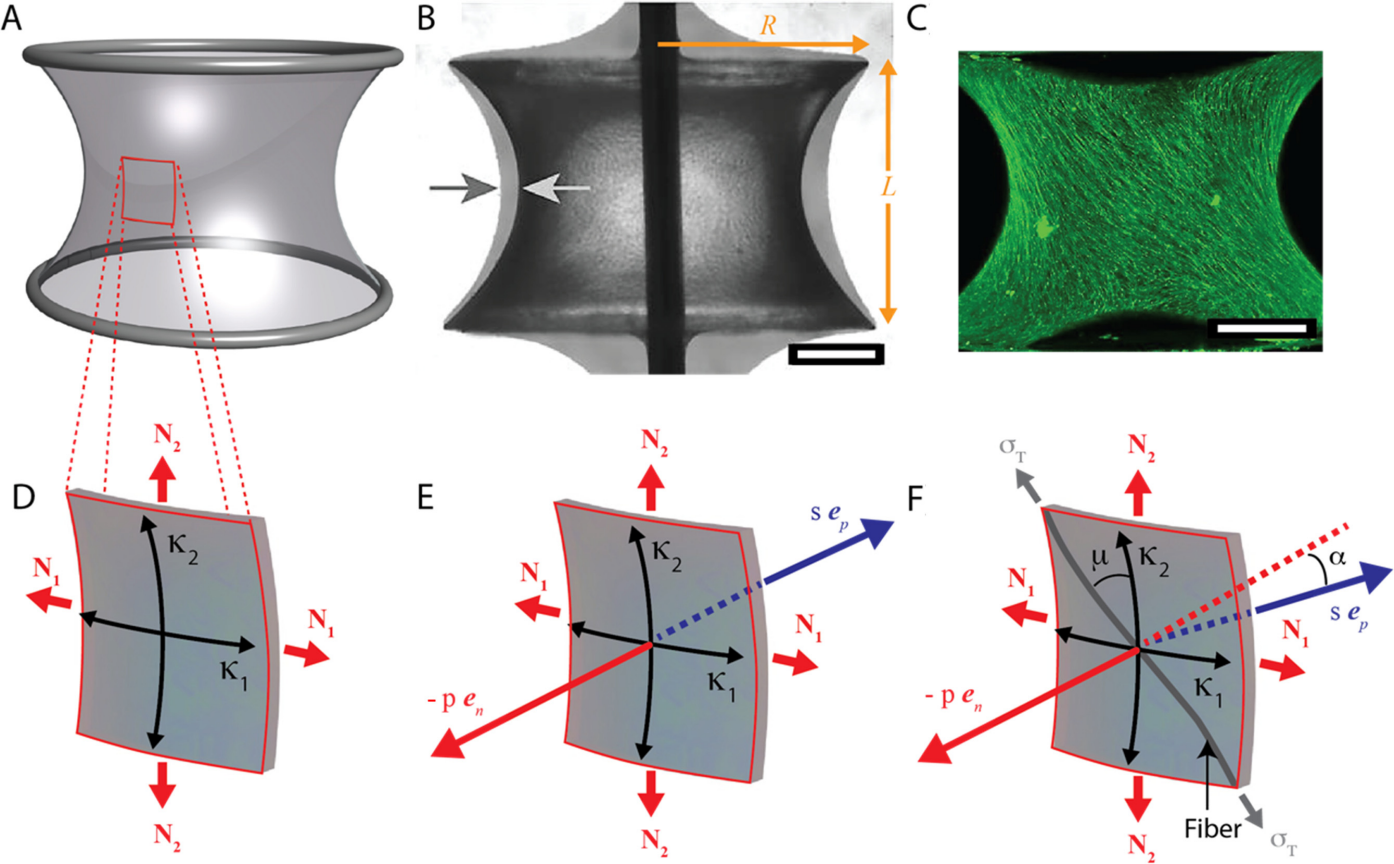
Experimental observations and definition of surface coordinates. (A) Image of a catenoid, i.e., the surface of revolution of a catenary, which satisfies Young–Laplace equation for zero pressure difference over the membrane. (B) Projection of bone-like tissue grown on a polymeric surface of revolution (capillary bridge) fixed on a central pin (dark line). The light arrow indicates the boundary of the polymeric surface, and the dark arrow indicates the position of the tissue after 30 days growth. $R$ and $L$ are the radius and separation of two circular disks corresponding to the upper and the lower boundary of the tissue. (C) Projection of a 3D light sheet fluorescence microscopy image of tissue stained for actin (green fibers). Note the strong orientation of the actin stress fibers. (D) The Young–Laplace equation (Eq. 1) can be understood by the tension balance over a surface patch, with two principal curvatures ${{\mathrm{\kappa }}}_1$ and ${{\mathrm{\kappa }}}_2$ and membrane forces ${N}_1$ and ${N}_2$ Eq. 2. (E) The pressure $p$ of the fluid within the volume generates a force directed along the normal to the surface ${{\boldsymbol{e}}}_n$. For isotropic mechanical surface properties, the resultant membrane force $s$ is also perpendicular to the surface but pointing in opposite direction $ - {{\boldsymbol{e}}}_n$. There is equilibrium if $s$ and $p$ have the same magnitude. (F) If the local mechanical response of the membrane is only generated by a fiber on the surface with tension ${{\mathrm{\sigma }}}_T$, then the resultant local force $s$ lies along ${{\boldsymbol{e}}}_p$, within the osculating plane of the fiber, and is not necessarily colinear with $p$ (i.e., 𝛼 ≠ 0). Therefore, the conditions for equilibrium of the surface are (i) that the direction of $p$ (i.e., ${{\boldsymbol{e}}}_n$) lies within the osculating plane of the curve describing the fiber (i.e., the angle 𝛼 = 0), and (ii) that the magnitudes of $s$ and $p$ are the same, which correspond to Eqs. 4a and 3, respectively. The fibril angle $\mu $ is measured between the fiber direction and ${N}_2$. The images in panels B and C are reproduced from ref. [14], under the CC BY-NC licence.

Surface energy concepts have been successfully applied especially to the development of epithelial layers, which are naturally two-dimensional tissues [6, 7]. However, it has also been shown that in vitro grown microtissues based on connective-tissue-forming fibroblasts develop a contractile layer at the surface of the growing tissue, where cells temporarily turn into myofibroblasts [13] capable of generating surface stresses. The shape of the resulting microtissues is fully compatible with constant curvature surfaces (Fig. 1A). Recently, it was shown that microtissues with cylindrical symmetry grown in vitro evolve into shapes with cylindrical symmetry and constant mean curvature [14], known as Delaunay surfaces [15, 16] (Fig. 1B). This is to be expected from the Young–Laplace equation that assumes isotropic, in-plane surface mechanical properties. However, the same recent work also revealed a puzzling observation that remains unexplained: Fluorescently stained, contractile actin filaments in the microtissue surface turn out to be highly aligned and follow helical paths around the tissue [14] (Fig. 1C) and indicate, therefore, that the surface does not have isotropic in-plane properties.

Thin elastic sheets reinforced by filaments are a well-known structural motif in biological materials, e.g., in human skin [17]. A well-studied case is the mechanical behavior of blood vessels that are subjected to blood pressure. Collagen fibril winding around the vessel tube has a strong impact on the elastic behavior of an artery [18]. A geometrically rather similar structure is the plant cell wall, where cellulose fibrils are winding within the cell wall around the tube-like cell [19]. The spiral angle of the cellulose microfibrils is known to have a decisive effect on the mechanical behavior of wood, for example [20]. All these examples refer to passive fibers (collagen or cellulose) that control the deformation for a material subjected to external forces, such as blood pressure in the artery or water swelling of the plant cell wall. In contrast, actin filaments in the near-surface region of a growing tissue [14] are actively contracting and, therefore, generating surface stress states in equilibrium with pressure in the volume of the growing tissue. Depending on geometry, contractile actin fibers near the surface region of the tissue might either increase or decrease the pressure inside the tissue. Based on the hypothesis that a reduction of pressure inside the growing tissue favors growth while an increase would hinder it [10], it will be interesting to explore which shapes emerge under the influence of contractile fibers on the surface.

In this paper, we first derive a simple extension of the Young–Laplace law, assuming that the surface properties are extremely anisotropic, where all the surface loads are carried by the fibers under tension. Note that this type of anisotropy is not the same as what is studied for the surface of crystals [21] or liquid crystals [22]. In those cases, the underlying bulk material itself has anisotropic properties. We also assume that the fibrous reinforcement remains bound to the surface in contrast to growing fibers constrained within a volume [23]. The case that we consider is inherently simpler and consists of an isotropic fluid bounded by a surface layer, modeled as a shell that consists of locally parallel fibers. Considering that the isostatic pressure inside the fluid is in equilibrium with the force in the surface fibers, the Young–Laplace equation is shown to be replaced by two simple conditions. We then consider the special case of volumes with rotational symmetry around an axis and derive the corresponding equilibrium shapes that are distinct from Delaunay surfaces corresponding to isotropic surface properties. The resulting shapes all show spiral arrangements of fibers on the surface, and for some special situations, the tissue shapes are qualitatively close to Delaunay shapes. This is finally discussed in terms of the known experimental observations [14].

## Modification of the Young–Laplace equation for a fiber surface

### Surface stress and pressure

The Young–Laplace equation was derived by both Young and Laplace in 1805 [1, 2]. It describes how the pressure difference acting on the surface of a thin shell (e.g., a drop), $p$, is related to the surface stress ${\gamma }_s$ [or surface tension, dimension (N/m)] via the prominent relation

(1) \begin{eqnarray*}
p\ = \ - {{\mathrm{\gamma }}}_s\ \left( {1/{R}_1 + 1/{R}_2} \right) = \ - 2{{\mathrm{\gamma }}}_sH.
\end{eqnarray*}

The surface stress ${\gamma }_s$ may also consist of several contributions, (see e.g., [22, 24]). Since ${\gamma }_s$ is assumed to be independent of any direction, often the term “isotropic surface stress” is used.

Both quantities ${R}_1$ and ${R}_2$ in Eq. 1, are the signed principal radii of curvature; the expression $( {1/{R}_1 + 1/{R}_2} )$ is thus twice the mean curvature $H$. This equation is a special formulation of the equilibrium equation for a membrane (see e.g., [25–29]) via the signed principal curvatures $1/{R}_1$ and $1/{R}_2$ with corresponding membrane forces ${N}_1$ and ${N}_2$, reading as

(2) \begin{eqnarray*}
p\ = \left( {\frac{{{N}_1}}{{{R}_1}} + \frac{{{N}_2}}{{{R}_2}}} \right).
\end{eqnarray*}

${N}_1$ and ${N}_2$ are the tensile membrane forces measured in the direction of the orthogonal principal curvature lines, denoted by the unit vectors ${{\boldsymbol{e}}}_1$ and ${{\boldsymbol{e}}}_2$. The quantity $p$ is the pressure difference across the membrane measured in the direction of ${{\boldsymbol{e}}}_1 \times \ {{\boldsymbol{e}}}_2 = {{\boldsymbol{e}}}_3\ $. This equilibrium equation is general and does not rely on the symmetry of any particular configurations, such as axisymmetric and spherically symmetric shells. It should be mentioned that ${N}_1$ and ${N}_2$ instead of the constant surface stress ${\gamma }_s$ were sometimes denoted in the literature as “anisotropic surface tensions” e.g., [30]. From the point of view of the mechanics of materials, the term “anisotropic” is, however, assigned to the material behavior.

The Young–Laplace equation (Eq. 1) has motivated the mathematical community to look for surfaces with constant mean curvature $H$, and thus constant pressure difference $p$. In 1841, Delaunay showed that the surfaces of revolution, namely; spheres, cylinders, nodoids, catenoids, and unduloids, have constant mean curvature $H$ and thus satisfy the Young–Laplace equation [15]. The Young–Laplace equation has also been previously used to describe tissue growth [31].

### In-plane anisotropic properties

Here we consider an isotropic fluid bounded by a surface layer modeled as a thin, unloaded shell reinforced with long contractile fibers. A pressure difference $p$ is generated across this surface layer by the fiber contraction, such as the actin stress fibers in the tissues shown in Fig. 1C [14]. Static equilibrium enforces that the direction ${{\boldsymbol{e}}}_T$ of any fiber is colinear with the (local) resultant membrane force (due to ${N}_1$ and ${N}_2$) in the tangent plane to a material point of the shell. We further define the local signed curvature $1/{R}_T$ of the fiber in direction of ${{\boldsymbol{e}}}_T$. It becomes immediately clear that for equilibrium the fiber needs to fulfill two conditions (Fig. 1F). The first one is due to the fact that the force resulting from a tension on the fiber will be within the osculating plane of the fiber at this point. This force (denoted $s$ in Fig. 1F) will be along the principal normal to the curve, ${{\boldsymbol{e}}}_p$, a unit vector perpendicular to the curve and pointing toward the local center of curvature and, therefore, lying within the osculating plane of the curve. The pressure difference across the surface results in a force that acts along the normal direction to the surface ${{\boldsymbol{e}}}_n$. In general, this is not parallel to the principal normal to the curve representing the fiber at the same point (Fig. 1F). Therefore, mechanical equilibrium requires that ${{\boldsymbol{e}}}_n = {{\boldsymbol{e}}}_p{\boldsymbol{\ }}$. The second condition links the magnitude of the fiber load with the pressure difference across the surface. A representative membrane force ${\sigma }_T \cdot d$ is assigned to the shell/fiber system with an average thickness $d$ and an average load stress ${{\mathrm{\sigma }}}_T$. According to Eq. 2, the local equilibrium between the fiber–shell system and the pressure difference $p$ enforces

(3) \begin{eqnarray*}
p\ = \frac{d}{{{R}_T}}\ {{\mathrm{\sigma }}}_T.
\end{eqnarray*}

Indeed, the membrane force perpendicular to the fiber is zero, and along the fiber, it is $d\ {{\mathrm{\sigma }}}_T$, so that Eq. 2 reduces directly to Eq. 3. Given that the pressure inside the volume is constant (as it should be for an isotropic fluid) and that the load along a fiber should also be constant, the requirement for a constant mean curvature that results from the Young–Laplace equation (Eq. 1) needs to be replaced by two conditions:

(4a) \begin{eqnarray*}
{{\boldsymbol{e}}}_n = {{\boldsymbol{e}}}_p,
\end{eqnarray*}

 

(4b) \begin{eqnarray*}
{R}_T = \ {\mathrm{constant}}{\mathrm{.}}
\end{eqnarray*}

The first condition ensures that the resultant force on the volume generated by the fiber tension is parallel to the surface normal (and, thus, able to compensate the internal pressure). The second condition ensures that the force along the fiber is constant.

Equation 4a states that the geodesic curvature of the line in the surface is zero. This means that contractile fibers have to follow geodesic lines on the surface. This is related to the well-known fact that an elastic strap stretched between two points over a curved surface follows a geodesic line because this corresponds to the shortest distance between the two points and, therefore, to the minimum of elastic strain energy of the strap. Equation 4b then states that the normal curvature of the lines (noting that their geodesic curvature is zero) must be constant in order to accommodate the constant pressure inside the body.

### Fiber-supported surfaces of revolution—generalization of delaunay surfaces

In order to get a geometric understanding of the requirements of Eqs. 4a and b, we analyze surfaces of revolution that fulfil these conditions. Since the experiment data reported in Fig. 1 were also obtained with tissues growing on surfaces of revolution, this will allow us a direct comparison with these experiments. In principle, however, the generalization of constant mean curvature surfaces as defined by Eqs. 4a and b does not need to be rotationally symmetric, depending on boundary conditions.

### Calculating the equilibrium for fibers on surfaces of revolution

We consider a surface of revolution $X$ given in a Cartesian coordinate system by the coordinates $x,\ y,\ z$ as products of the shape function $g( z )$ and the polar angle $\theta $ in the $x\hbox{--}y$ plane. Moreover, we assume that this surface consists of fibers that are positioned around the $z$-axis according to a function $\theta \ = \ \theta ( z ) + {\theta }_0$. The angle ${\theta }_0$ at $z\ = \ 0$ indicates the starting point of any particular fiber. Making use of the rotational symmetry, we restrict our analyses to the fiber where $\theta \ ( 0 ) = \ 0$. Therefore, we can describe the surface of the system in vector form $X( {{\mathrm{\theta }},z} )$ depending on only two coordinates, $\theta $ and $z$.

(5) \begin{eqnarray*}
X\ \left( {\theta ,z} \right) = \left( {\begin{array}{@{}*{1}{c}@{}} x\\ y\\ z \end{array}} \right){\mathrm{\ }} = \left( {\begin{array}{@{}*{1}{c}@{}} {g\left( z \right){\mathrm{cos}}\theta }\\ {g\left( z \right){\mathrm{sin}}\theta }\\ z \end{array}} \right)\ .
\end{eqnarray*}

Using standard differential geometry (see e.g., [32, 33]), we can determine the unit normal vector of the fiber ${{\boldsymbol{e}}}_p$ (that is, the unit normal vector lying within the osculating plane and along which the resulting force onto the surface will be directed).

(6) \begin{eqnarray*}{{\boldsymbol{e}}}_p = \frac{1}{{\sqrt {{{x^{\prime\prime}}}^2 + {{y^{\prime\prime}}}^2 + {{\left( {y^{\prime\prime}x^{\prime} - x^{\prime\prime}y^{\prime}} \right)}}^2} \sqrt {{{x^{\prime}}}^2 + {{y^{\prime}}}^2 + 1} }}{\mathrm{\ }} \cdot \left( {\begin{array}{@{}*{1}{c}@{}} {x^{\prime\prime}\left( {1 + y{{\mathrm{^{\prime}}}}^2} \right) - y^{\prime\prime}x^\prime y^{\prime}}\\ {y^{\prime\prime}\left( {1 + x{{\mathrm{^{\prime}}}}^2} \right) - x^{\prime\prime}x^\prime y^{\prime}}\\ { - \left( {x^\prime x^{\prime\prime} + y^\prime y^{\prime\prime}} \right)} \end{array}} \right).\end{eqnarray*}

Note that primes refer to derivatives with respect to $z$. Likewise, the unit surface normal, ${{\boldsymbol{e}}}_n$, is given by

(7) \begin{eqnarray*}
{{\boldsymbol{e}}}_n = \frac{1}{{\sqrt {{x}^2\left( {1 + {{x^{\prime}}}^2} \right) + {y}^2\left( {1 + {{y^{\prime}}}^2} \right) + 2xyx^\prime y^{\prime}} }}\ \left( {\begin{array}{@{}*{1}{c}@{}} x\\ y\\ { - \left( {xx^{\prime} + yy^{\prime}} \right)} \end{array}} \right).
\end{eqnarray*}

The curvature, $\ {\kappa }_T = \ 1/{R}_T$, of the fiber is given by

(8) \begin{eqnarray*}
{\kappa }_T = \frac{{\sqrt {{{x^{\prime\prime}}}^2 + {{y^{\prime\prime}}}^2 + {{\left( {y^{\prime\prime}x^{\prime} - x^{\prime\prime}y^{\prime}} \right)}}^2} }}{{{{\left( {{{x^{\prime}}}^2 + {{y^{\prime}}}^2 + 1} \right)}}^{3/2}}}.
\end{eqnarray*}

As outlined above, the vector ${{\boldsymbol{e}}}_p$ is assumed to be parallel to the surface normal ${{\boldsymbol{e}}}_n$. It follows that

(9) \begin{eqnarray*}
{{\boldsymbol{e}}}_p \times {{\boldsymbol{e}}}_n \equiv 0.
\end{eqnarray*}

Including the condition of constant fiber curvature Eq.8 and rearranging gives a set of two differential equations in $x,y$, as

(10a) \begin{eqnarray*}
x^{\prime\prime} = {\kappa }_T\ \frac{{\left( {x\left( {1 + {{x^{\prime}}}^2} \right) + yx^\prime y^{\prime}} \right)\left( {1 + {{x^{\prime}}}^2 + {{y^{\prime}}}^2} \right)}}{{{{\left( {{x}^2\left( {1 + {{x^{\prime}}}^2} \right) + 2xyx^\prime y^{\prime} + {y}^2\left( {1 + {{y^{\prime}}}^2} \right)} \right)}}^{1/2}}},
\end{eqnarray*}

 

(10b) \begin{eqnarray*}
{\mathrm{y^{\prime\prime}}} = {\kappa }_T\ \frac{{\left( {1 + {{x^{\prime}}}^2 + {{y^{\prime}}}^2} \right)\left( {xx^\prime y^{\prime} + y\left( {1 + {{y^{\prime}}}^2} \right)} \right)}}{{{{\left( {{x}^2\left( {1 + {{x^{\prime}}}^2} \right) + 2xyx^\prime y^{\prime} + {y}^2\left( {1 + {{y^{\prime}}}^2} \right)} \right)}}^{1/2}}}.
\end{eqnarray*}

The fiber curvature, ${\kappa }_T$, can be positive or negative (see Supplementary Information for a full derivation). It is helpful to rewrite Eqs. (10a andb) in cylindrical coordinates as

(11a) \begin{eqnarray*}
g^{\prime\prime} = \frac{{1 + {{g^{\prime}}}^2}}{{g\ \left( {K{g}^2 - 1} \right)}}\ \left( {1 - K\ {\kappa }_T\ {g}^3\sqrt {1 + {{g^{\prime}}}^2} } \right),
\end{eqnarray*}

 

(11b) \begin{eqnarray*}
\theta ^{\prime} = \frac{1}{g}\ \sqrt {\frac{{1 + {{g^{\prime}}}^2}}{{K{g}^2 - 1}}} .
\end{eqnarray*}

The constant $K$ is an integration constant that depends on boundary conditions. Note that both equations above have symmetric solutions where $g\ ( { - z} ) = \ g( z )$ and $\theta \ ( { - z} ) = \ - \theta ( z )$. It is worth noting that  Eq. 11b is the classical equation for geodesic lines on a surface of revolution [34].  Equation 11a results from the condition that the geodesic should have constant normal curvature $\ {\kappa }_T$. Denoting α the angle between the geodesic line and the meridian of the surface of revolution at the same point of the surface, Clairaut’s relation [34] must hold: $g( z )\ \sin \alpha = 1/\sqrt K \ $. This means that the angle $\alpha $ does not depend on $\theta $. Therefore, families of geodesics are spiraling around the axis of revolution in a parallel fashion.

### Boundary conditions

**Fig. 2.**
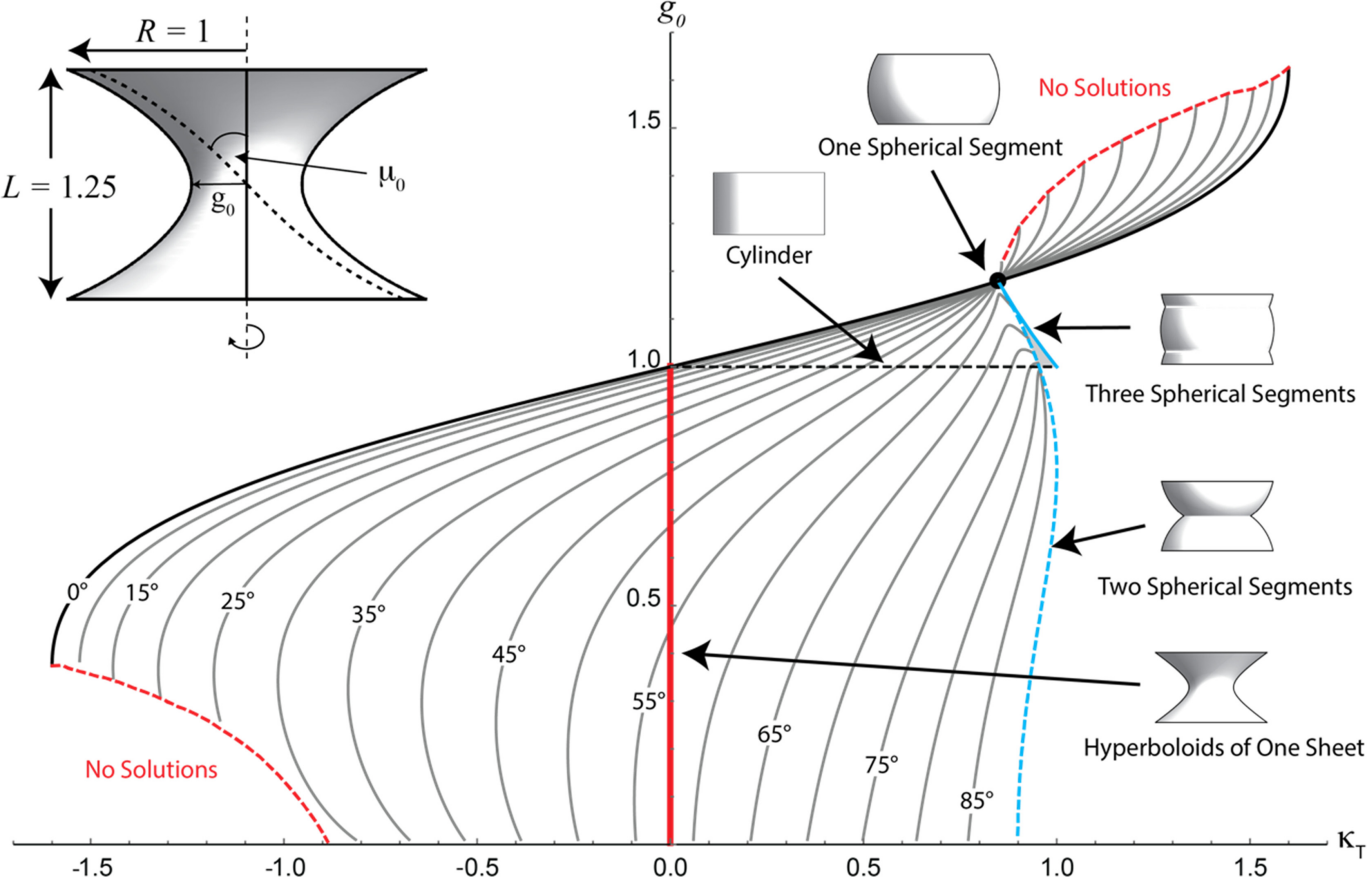
Surfaces of revolution satisfying Eqs. 11a and b and the boundary conditions 14 as indicated in the inset top left, characterized by the (constant) curvature ${{\mathrm{\kappa }}}_{\mathrm{T}}$ of the fibers which stabilize the shape and the neck radius ${{\mathrm{g}}}_0$. Fibers follow spiraling paths, and the fibril angle at the equator ${{\mathrm{\mu }}}_0$ is indicated for each set of curvature and neck radius. The inset (top-left) shows one such surface of revolution consisting of fibers of constant curvature that cross the equator with a “microfibril” angle, ${{\mathrm{\mu }}}_0$. The dashed line on the inset indicates the path of one such fiber. Grey lines in the main diagram give the relationship between ${{\mathrm{g}}}_0$ and ${{\mathrm{\kappa }}}_{\mathrm{T}}$ for a fixed fibril angle ${{\mathrm{\mu }}}_0$ at the equator. The dashed red lines indicate the limits below or above which no solutions can be found. The solid red line shows the range of solutions satisfied by hyperboloids of one sheet, the dashed black line shows the range of cylindrical solutions. The full black circle indicates the solution given by one spherical segment. All solutions with microfibril angles less than 58° pass through this point. The dotted blue line indicates the relationship between ${{\mathrm{g}}}_0$ and ${{\mathrm{\kappa }}}_{\mathrm{T}}$ or a stack of two spherical segments, and the solid blue line for a stack of three spherical segments. Note that these solutions are not differentiable at the joint between the spherical segments and can be considered as limit cases. The lower part of the graph (${{\mathrm{g}}}_0$ < 1) corresponds to necked structures as shown in the inset. The upper part of the graph describes bulged structures (${{\mathrm{g}}}_0$ > 1), akin to the single spherical segment. The light grey region between the dotted blue line and the solid blue line is shown in more detail in the supplementary information (Fig. S2).

We consider surfaces of revolutions bounded by two circles with radius $g\ ( { \pm L/2} ) = \ R$ at the positions $z\ = \ \pm L/2$, as sketched in Fig. 2 (upper left corner). The neck radius at $z\ = \ 0$ is defined as $g\ ( 0 ) = {g}_0\ $, and we search for symmetric solutions with respect to the axis of revolution yielding $g^{\prime}\ ( 0 ) = \ 0$. We introduce the fiber angle ${\mu }_0$ between the $z$-direction and the fiber at the neck ($z\ = \ 0$) (see Figs. 1F and  2), which is given by ${\mathrm{cos}}\ {\mu }_0 = \ 1/\sqrt {1 + g_0^2\ {{( {\theta ^{\prime}( 0 )} )}}^2} $. With this definition, it follows from  Eq. 11b (taken at $z\ = \ 0$) that the integration constant $K$ can be written as

(12) \begin{eqnarray*}
K\ = \frac{1}{{g_0^2{{\sin }}^2{\mu }_0}}.
\end{eqnarray*}

Clairaut’s relation then gives

(13) \begin{eqnarray*}
\sin \alpha = \frac{{{g}_0}}{{g\left( z \right)}}\ \sin {\mu }_0.
\end{eqnarray*}

This relation reflects the fact that, at the neck ($z\ = \ 0)$, the meridian of the surface is parallel to the axis of revolution. The boundary conditions sketched in Fig. 1 imply that $g\ ( {L/2} ) = \ g\ ( { - L/2} ) = \ R$. In the experimental setting shown in Fig. 1, we set $2L/R\ = \ 1.25$. The symmetry condition and the value ${g}_0$ uniquely define the surface. Each fiber line is then contained in this surface and crosses the neck at $\theta ( 0 )$ with an angle ${\mu }_0$. Due to axisymmetry we only need to consider fibers for which $\theta \ ( 0 ) = \ 0$.

Equation 11a is a second order nonlinear differential equation, and 11b is a first order nonlinear differential equation meaning that for any value of ${\kappa }_T$ we need a total of two boundary conditions for $g$ and one for $\theta $ as

(14) \begin{eqnarray*}
g^{\prime}\,\,( 0 ) = 0, g(0) = {g}_{0},\theta(0) = 0
.
\end{eqnarray*}

### A numerical study

It turned out to be more efficient than solving the equations numerically for fixed ${\kappa }_T$, to only fix ${g}_0$ and ${\mu }_0$ over a range of ${\kappa }_T$. Solutions were accepted that satisfied the additional constraint $g\ ( {L/2} ) = \ R$, thereby giving the value of ${\kappa }_T$ in accordance with these conditions. Solving Eqs. 11 to 14 for surfaces of revolution, we find typically either neck-shaped or barrel-shaped surfaces bounded on the upper and the lower side by disks or radius *R*. The results are summarized in Fig. 2 (see also Figs. S1 and S2). To simplify the graphs, we choose a coordinate system so that $R\ = \ 1$ and, consequently, $L\ = \ 1.25$.

The solutions include expected shapes, such as the sphere, the cylinder, and the hyperboloid. The latter is obtained by straight fibers and is found in the graph at ${\kappa }_T = \ 0$. Different fibril angles correspond to different neck radii. At the fibril angle of ${\mu }_0 = \ 0$, the hyperboloid coincides with a cylinder, as expected. For positive values of ${\kappa }_T$ solutions tend toward stackings of spherical segments that satisfy Eq. 13, except at the joint (see Supplementary Information for more detail). Finally, the upper black curve for ${\mu }_0 = \ 0$, corresponds to segments of a torus.

All shapes in Fig. 2 will be barrel-like in the upper part where ${g}_0 > 1$ and neck-like in the lower half, where ${g}_0 < 1$. All shapes on the left side (${\kappa }_T < 0$) correspond to a situation where tensed fibers will create a negative pressure inside the volume (according to Eq. 3), while for all shapes on the right side of the figure (${\kappa }_T > 0$) fiber tension will create a positive pressure. In the case of a growing tissue, negative pressure can be interpreted as a support to the volume increase and, therefore, growing of the tissue, while positive pressure created by the fibers will rather slow down the tissue growth.

**Fig. 3.**
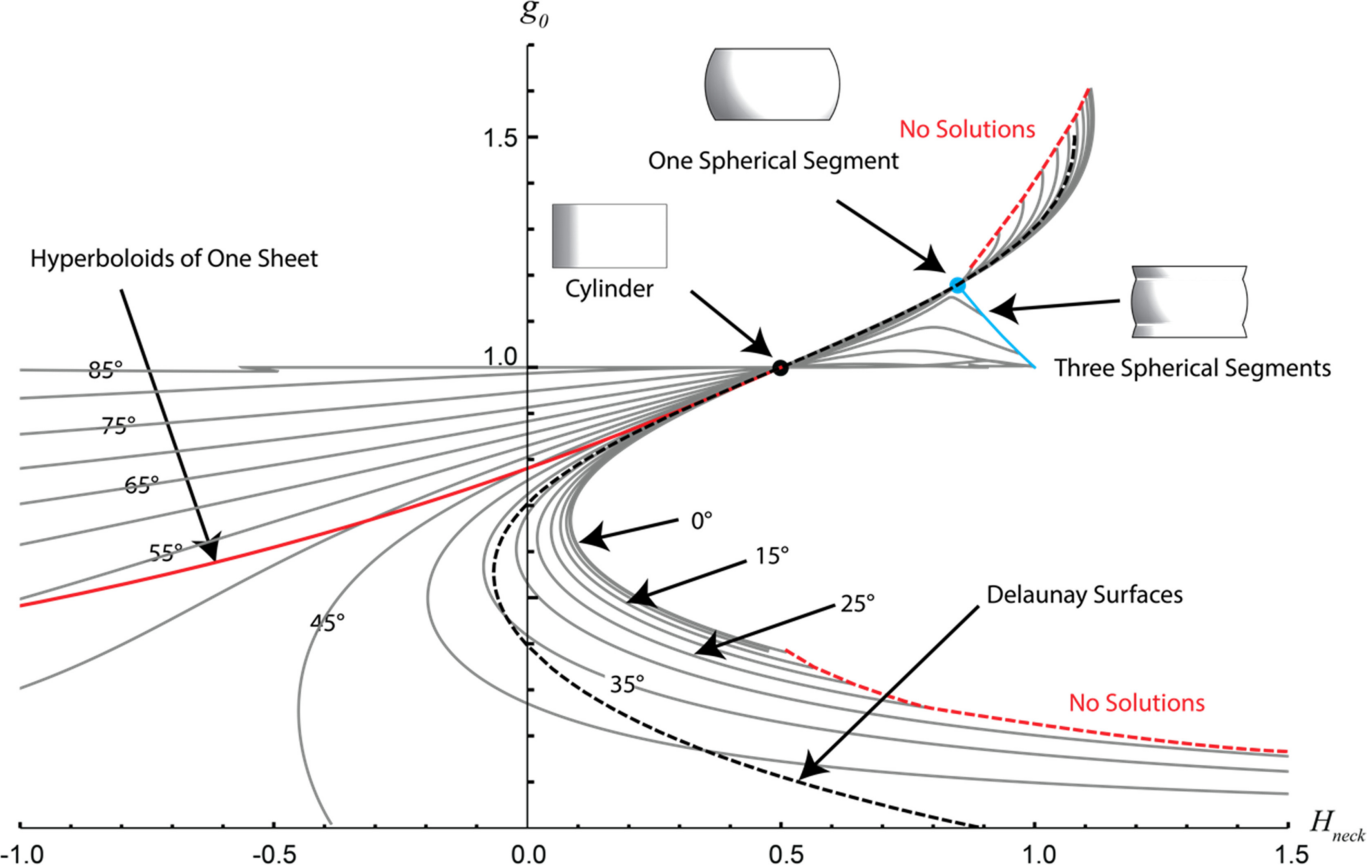
Alternative representation of Fig. 2, showing the neck radius, ${g}_0$, versus the mean curvature at the neck, ${H}_{neck}$, for different “mifrofibril” angle, ${\mu }_0$, at the equator. Grey lines give solutions in which the microfibril angle at the neck is fixed. The dashed red lines indicate the limits beyond, which no solutions can be found. The solid red line shows the range of solutions satisfied by hyperboloids of one sheet, the solid blue line indicates solutions for stacks of three spherical segments. The black dot indicates cylindrical solutions, the blue dot shows the solution for one segmented sphere. The dashed black line shows the relationship between neck radius and mean curvature for Delaunay surfaces that satisfy the boundary conditions. Note that this curve is close to the solutions for ${\mu }_0$ = 35° for the neck ranging from about ${{\mathrm{g}}}_0$ = 0.4 to ${{\mathrm{g}}}_0$ = 0.7.

For a better comparison with Delaunay surfaces, we redraw Fig. 2 by using the mean curvature at the neck for the *x*-axis, instead of the fiber curvature (Fig. 3). Note that the mean curvature is not constant for the shapes considered here, while it is constant for the Delaunay surfaces (Fig. S4). The Delaunay surfaces are indicated in Fig. 3 by a dotted line. It is quite remarkable that this dotted line is quite close to line corresponding to ${\mu }_0 = \ 35^\circ $ in the range of parameters from ${g}_0$ = 0.4 to ${g}_0$ = 0.7. This means that surfaces stabilized by fibers with this fibril angle will be close to Delaunay surfaces.

### Implications for the interpretation of experiment data

**Fig. 4.**
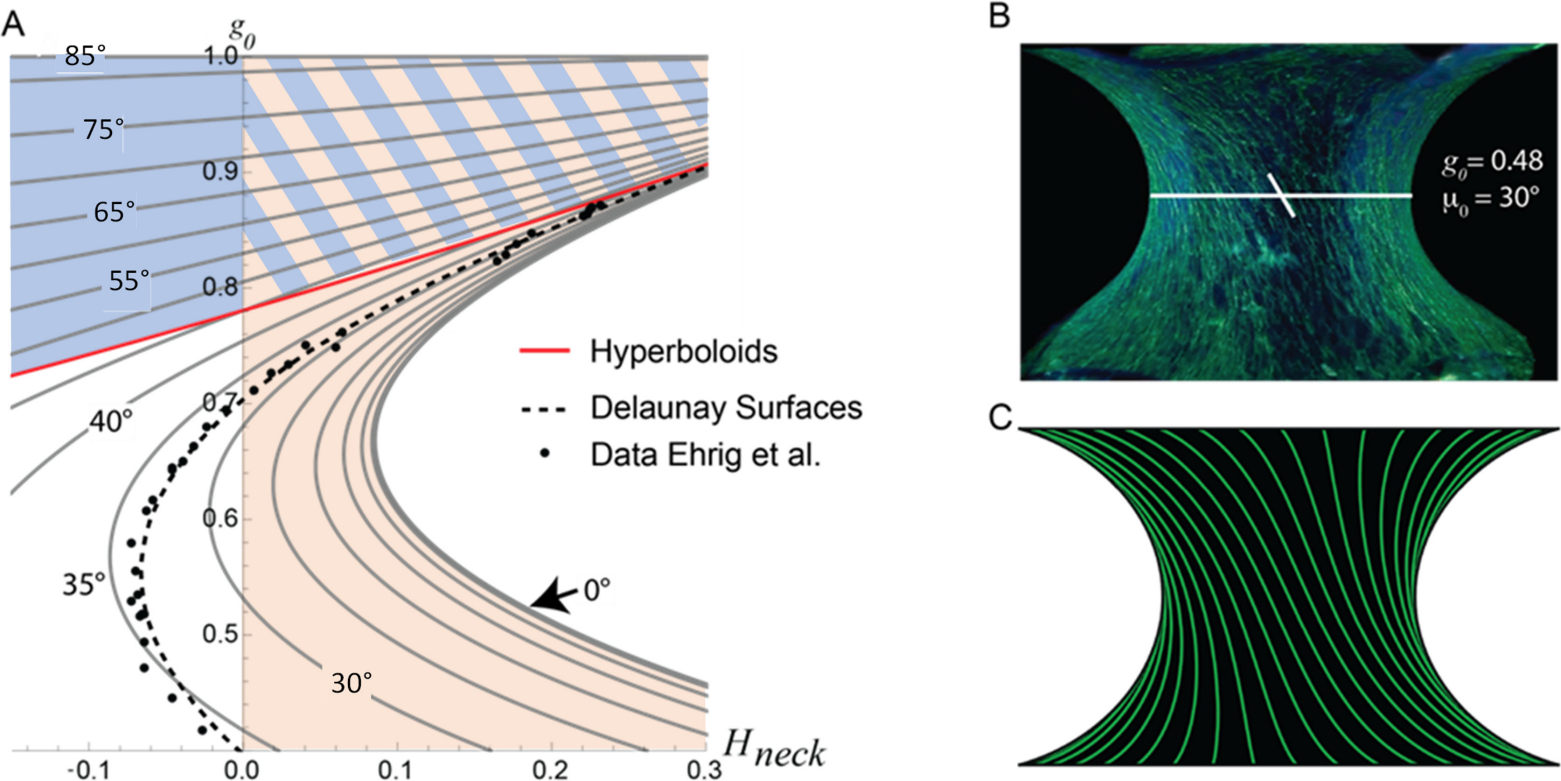
Comparison of the model based on anisotropic surface contraction to the experiments of ref. [14]. Panel A is an enlargement of a portion of Fig. 3 with dots indicating all the tissue growth experiments performed in ref. [14]. It is quite remarkable that these dots are simultaneously close to the line for Delaunay surfaces (dotted line) and to fiber-stabilized volumes with a microfibril angle at the neck between 30° and 35°. It is also important to realize that the size of these dots is not representative for the experimental uncertainty in measuring curvatures. To account for this, they should be much larger and make the figure unreadable. Note that the induced tissue pressure is positive for all configurations in the blue area above the red line that corresponds to hyperboloids. Below this line, the tissue pressure induced by contraction of the fibers is negative and favors tissue growth. For isotropic mechanical surface properties, the surface stress induces a positive tissue pressure on the right side of the vertical line with zero mean curvature (sepia area in panel A). Panel B shows a typical microtissue grown in vitro under the boundary conditions used in our model calculations. The green coloration is due to actin staining and the microfibril angle in the neck is around 30° to 35° for most of the experiments. Panel C shows a solution to Eq. (11) with predicted fiber paths shown in green and a microfibril angle at the neck of 30°.

We now turn back to the experiments that motivated the current study and compare the published experiment data with our model (Fig. 4). Experimental values, obtained from [14], of neck radius versus curvature are indicated by dots in this figure. The data were originally interpreted as being compatible with Delaunay surfaces (dashed line Fig. 4A). For a better comparison, we therefore represent the graph as neck radius versus mean curvature at the neck, ${H}_{neck}$. Indeed, mean curvature is constant for a Delaunay surface and $H \equiv {H}_{neck}$ The solutions of Eq. 11 (where $H$ is not a constant) are also plotted on this graph, whereby the *x*-axis represents ${H}_{neck}$. In order to interpret this graph, it is worth going back to Eq. 1 and 3. In case of isotropic surface properties (and a resulting Delaunay curve) the surface stress reduces the pressure in the volume for negative ${H}_{neck}$ (that is on the left of the graph in Fig. 4A). For positive ${H}_{neck}$ (area highlighted in sepia in Fig. 4A), the surface tension increases the pressure in the tissue and, therefore, hinders its growth. This is in stark contrast to the fact that no fundamental change in tissue growth was found experimentally [14] when the neck radius ${g}_0$ started to exceed the value of about 0.7 (which is the point where the mean curvature starts to be positive, see Fig. 4A).

In contrast to this fact, contractile fibers decrease the pressure for all neck radii below the red line in Fig. 4A. Only in the region highlighted in blue, contractile fibers would increase the pressure in the volume and, therefore, hinder rather than favor tissue growth. Very interestingly, all experimental data points in Fig. 4A are located in the area where contractile fibers favor tissue growth.

**Fig. 5.**
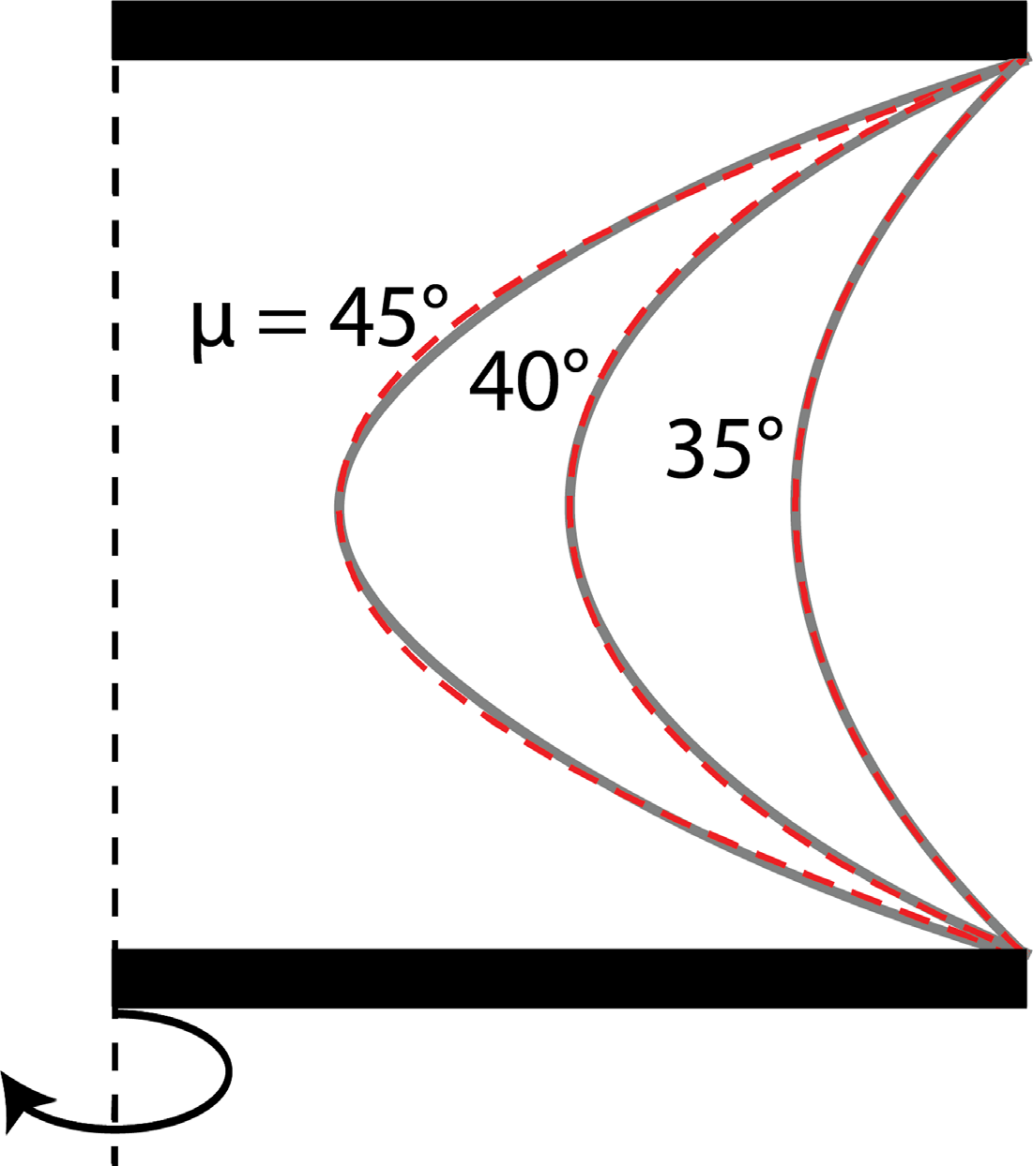
Comparison of Delaunay shapes (red) for different values of neck radii (0.25, 0.5, and 0.75) with solutions of Eq. 11 (grey lines) using values of the fiber angle at the neck, ${\mu }_0$, as indicated. While the surfaces are mathematically distinct, they are essentially indistinguishable in a typical tissue culture experiment. A more detailed comparison is shown in Fig. S4.

Moreover, it turns out that the solutions of Eqs. 11a and b can hardly be distinguished experimentally from Delaunay surfaces, in case that the fiber angle at the neck is in the range between 35° and 45°. Figure 5 shows differences between these two types of surfaces that are, indeed, too small to be distinguished in tissue culture experiments. Finally, it is striking to see that the actin stress-fiber angle that was measured in the experiments (Fig. 4B) is, indeed, in the range of 35° to 40°. The fiber paths predicted from Eqs. 11a and b for a tilt angle at the neck of 35° (Fig. 4C) also match remarkably well the experimental images.

This observation indicates that tension generated by aligned stress fibers in cells of the near-surface region produces a reduction of pressure inside the tissue more effectively than in an isotropic surface stress state. Indeed, for the example studied, an isotropic surface stress state would generate negative pressure only up to a neck radius of ${g}_0 \approx 0.7$, while aligned stress fibers in the surface provide negative pressures up to a neck radius of ${g}_0 \approx 0.9$.

At this point, it remains unclear why cells chose an angle of approximately 35° to develop the stress fibers, but one may speculate, based on the fact that force generation by actin–myosin interaction is always intermittent [35], that in the relaxation phases of the contractile fibers the tissue relaxes to shapes dictated by the surface energy without contractile fibers. Based on the boundary conditions, these shapes would be Delaunay surfaces. As visible in Fig. 4A, contractile fibers have the same values of ${H}_{neck}$ and ${g}_0$ as the Delaunay surface when ${\mu }_0$ is close to 35°. It is then perhaps not unlikely that cells, when they develop fiber tension, chose an orientation of ∼35° both to preserve the actual shape and to provide additional negative pressure inside the tissue to allow for and to enhance growth.

The reason why a particular fiber chirality appears in the tissue growth experiments described in ref. [14] is currently unknown, and our model is equally valid for both possible chiral directions. Possibly, the inherent chirality of cells [36] may prefer one direction over another. Further experiments will be required to investigate this.

In addition, the contractile actin stress fibers will only have a significant effect if indeed, as assumed in the treatment above, the surface stress generated by the fibers is significantly larger than the isotropic surface tension of the tissue. The surface tension of tissues has been reported to be on the order of 0.1 to 3 mN/m [37, 38]. This tension arises due to a combination of cell–cell adhesion and cortical tension. In previous work [13], we have shown that the cells in the surface layer acquire the phenotype of myofibroblasts. Such cells were reported to generate tension on the order of 2 to 5 kPa [39]. Assuming that these cells occupy the top 10 μm below the surface of the tissue, this converts roughly to 20 to 50 mN/m, which would be up to two orders of magnitude larger than the surface tension. These rough estimates suggest that the contraction of actin stress fibers may, indeed, control the shape of the growing tissue.

The present theory is only geometrical, and the mechanical properties of the tissue (except assuming that it is fluid, with all shear moduli equal to zero) do not enter the description. This can only be justified if the remodeling (that generates fluidity) is faster than the volume change due to growth. Otherwise, the time-dependent properties of the fluid need to be modeled, which most likely requires numerical modeling beyond the analytical description presented here. Another limitation is the assumption that fibers do not transfer load laterally. Relaxing this assumption implies that the surface force would not anymore be unidirectional along a fiber, which considerably complicates the problem and would also likely require numerical analysis. Qualitatively, one can consider situations, where some load is transferred between neighboring fibers, to be intermediate between the model analyzed here and the classical treatment with isotropic surface tension. Finally, one should consider that individual fibers are not as long as it seems in Fig. 1, which means that they need to be assembled into larger load carrying bundles. In that context, it is interesting to notice that—although actin fibers are intracellular—the directions of stress fibers are consistent over distances much larger than the size of a cell [40]. This means that there must be some mechanical communication between cells that is implicit in the present model.

## Conclusion

The well-known Young–Laplace equation can be modified to describe the pressure change induced by parallel contractile fibers instead of surface stress states in a mechanically isotropic surface. The pressure inside the volume is then proportional to the stress in the fibers and, therefore, inversely proportional to the radius of curvature of the fibers. The directions of the contractile fibers represent geodesic lines of the surface. The surface shape results from the fact that the geodesic lines need to have constant normal curvature. The current analysis of surfaces of revolution fulfilling these conditions shows that such surfaces are mathematically different from constant mean curvature (Delaunay) surfaces. However, they might be numerically sufficiently similar to make them indistinguishable in tissue culture experiments.

## Supplementary Material

pgac292_Supplemental_File

## Data Availability

All data are available in the manuscript and the supplementary material.

## Preprints

A preliminary version of this manuscript was posted in preprint form on arXiv: https://arxiv.org/abs/2112.08502.
