# Supplementary material for: On shape forming by contractile filaments in the surface of growing tissues
Source: PNAS Nexus. 2022 Dec 12;2(1):pgac292. doi: 10.1093/pnasnexus/pgac292 (PMC9832972; doi:10.1093/pnasnexus/pgac292)
Supplement: pgac292_Supplemental_File [file pgac292_supplemental_file.pdf]

Supplementary Information for

On shape forming by contractile filaments in the surface of growing tissues.

Peter Fratzl[1], F. Dieter Fischer[2], Gerald A. Zickler[2], John W.C. Dunlop[3]

[1]Max Planck Institute of Colloids and Interfaces, Department of Biomaterials, Potsdam Science Park, 14476 Potsdam-Golm
[2]Montanuniversität Leoben, Institute of Mechanics, 8700 Leoben, Austria
[3]Morphophysics Group, Department of the Chemistry and Physics of Materials, University of Salzburg, 5020 Salzburg, Austria

*corresponding authors: Peter Fratzl and John W.C. Dunlop

Email: fratzl@mpikg.mpg.de, john.dunlop@plus.ac.at

This PDF file includes:

Supplementary text
Figures S1 to S4
Table S1

Supplementary Information Text

In the first section of the Supplementary Information, we show the full derivation of equations **10** and **11**. In the second section we explore in more detail the solution space of equations **11a** and **b**, and discuss specific limit cases as of the torus and when the surface of revolution become close to stacks of spherical segments. In the final section we compare solutions of our equations with the well-known Delaunay surfaces of revolution.

1) Derivations of Equations 10a,b and 11a,b

We give more details of the derivations in order to help the reader following the text. Equations **6-9** are repeated from the main text as follows:

$$\boldsymbol{e}_p = \frac{1}{\sqrt{x''^2+y''^2+(y''x'-x''y')^2}\sqrt{x'^2+y'^2+1}} \cdot \begin{pmatrix} x''(1+y'^2)-y''x'y' \\ y''(1+x'^2)-x''x'y' \\ -(x'x''+y'y'') \end{pmatrix}, \quad [6]$$

$$\boldsymbol{e}_n = \frac{1}{\sqrt{x^2\left(1+x'^2\right)+y^2\left(1+y'^2\right)+2xyx'y'}} \begin{pmatrix} x \\ y \\ -(xx'+yy') \end{pmatrix}, \quad [7]$$

$$\kappa_T = \frac{\sqrt{x''^2+y''^2+(y''x'-x''y')^2}}{\left(x'^2+y'^2+1\right)^{3/2}}, \quad [8]$$

$$\boldsymbol{e}_p \times \boldsymbol{e}_n \equiv \boldsymbol{0}. \quad [9]$$

Equation **9** will hold if the following equation is true:

$$\begin{pmatrix} x''(1+y'^2)-y''x'y' \\ y''(1+x'^2)-x''x'y' \\ -(x'x''+y'y'') \end{pmatrix} \times \begin{pmatrix} x \\ y \\ -(xx'+yy') \end{pmatrix} = \boldsymbol{0}. \quad [\text{S-1}]$$

This implies that:

$$\begin{pmatrix} -(y''(1+x'^2)-x''x'y')(xx'+yy')+(x'x''+y'y'')y \\ \left(-(x'x''+y'y'')\right)x+(x''(1+y'^2)-y''x'y')(xx'+yy') \\ (x''(1+y'^2)-y''x'y')y-(y''(1+x'^2)-x''x'y')x \end{pmatrix} = \boldsymbol{0}. \quad [\text{S-2}]$$

One can show that all terms in this equation are satisfied if the relation below is valid,

$$x''\left(y+y'(xx'+yy')\right) = y''\left(x+x'(xx'+yy')\right). \quad [\text{S-3}]$$

Rearranging equation **8** as follows:

$$x''^2+y''^2+(y''x'-x''y')^2 = {\kappa_T}^2\left(x'^2+y'^2+1\right)^3. \quad [\text{S-4}]$$

Equation **S-3** can then be solved for x'', and x'' inserted in equation **S-**4 gives:

$$y''^2\left(\left(\frac{x+x'(xx'+yy')}{y+y'(xx'+yy')}\right)^2+1+\left(x'-x\left(\frac{x+x'(xx'+yy')}{y+y'(xx'+yy')}\right)y'\right)^2\right) = {\kappa_T}^2\left(x'^2+y'^2+1\right)^3. \quad [\text{S-5}]$$

This can be readily solved for y'' yielding equations **10a** and **10b**.

$$x'' = \kappa_T \frac{\left(x\left(1+x'^2\right)+yx'y'\right)\left(1+x'^2+y'^2\right)}{\left(x^2\left(1+x'^2\right)+2xyx'y'+y^2\left(1+y'^2\right)\right)^{1/2}}, \quad [10a]$$

$$y'' = \kappa_T \frac{\left(1+x'^2+y'^2\right)\left(xx'y'+y\left(1+y'^2\right)\right)}{\left(x^2\left(1+x'^2\right)+2xyx'y'+y^2\left(1+y'^2\right)\right)^{1/2}}. \quad \textbf{[10b]}$$

It is useful to solve these equations also in cylindrical coordinates. To do this we rearrange equation **S3** as

$$x\,y'' - y\,x'' = (y\,y' + x\,x')(y'x'' - x'y''). \quad \textbf{[S-6]}$$

Using an intermediate variable, $h = g\theta'$, this equation can be rewritten as

$$(1 + g'^2)\left(\frac{h}{g} + \frac{h'}{g'}\right) - h\left(g'' - \frac{h^2}{g}\right) = 0. \quad \textbf{[S-7]}$$

This can be integrated giving

$$h = g\,\theta' = \sqrt{\frac{1+g'^2}{Kg^2-1}}. \quad \textbf{[S-8]}$$

K is an integration constant that can be linked to the fiber angle at the neck (Equation **13)**. Rearranging equation **S-8** directly leads to equation **11b**. Using this result together with Equation **S-4**, rewritten in cylindrical coordinates gives, after some rearranging, equation **11a**.

2) Analysis of the solution space of Equations 11a and b

A systematic numerical study of equations **11a** and **11b** was performed to explore the range of geometries of constant fiber curvature surfaces of revolution (Figs. 2, S1 and S2). For negative fiber curvatures the solutions all have profiles with surface curvatures of constant sign, with the profiles "pinching" together at a neck radius of zero. Surfaces with zero fiber curvature are simply ruled surfaces or hyperboloids of one sheet (red-line in Fig. 2). These surfaces have a maximum neck radius of 1 being the cylinder solution (Black dashed line in Fig S2). For cylinders the fiber curvature is given by $\kappa_T = \sin^2\mu_0/R$, with microfibril angles ranging from 0 to 90°. For positive fiber curvatures and small neck radii, the surface curvatures change sign, with local "concave" areas at the neck and smooth "convex" areas above and below (bottom right of Fig S1). For neck radii larger than 1, the solutions become more convex (top right of Fig S1). To further understand the solution space of the equations it is useful to look in more details at the plot of the neck radius and fiber curvature calculated from the numerical solutions of equations **11a** and **11b** (Fig S2). As the fiber curvature increases (towards the right of Fig. S1), we approach limits which correspond to stackings of two or more spherical segments. Although these are not differentiable at the joint between the spherical segments, these limit cases help us understand the geometry of the numerical solutions, which are smoothed versions of the spherical sphere stackings.

A fiber embedded into a spherical surface segment with the same radius of curvature as the fiber curvature, will satisfy equations **11a** and **11b**. This can be seen in the numerical solutions that tend towards spherical solutions for large values of fiber curvature. This means it is possible to construct via stacking of spherical segments new solutions that will also satisfy equations **11a** and **11b**, except at the cusp-like join. This can be better understood in Fig S2B, where the boxed region highlighted in Fig S2A is replotted showing some examples of limit cases consisting of stacked spherical segments. The simplest solution with a spherical segment is the single truncated sphere indicated by the black point in Fig S2B. All numerical solutions, found with fiber angles at the neck less than $\left(\arctan\frac{R}{L/2}\right)$ ~58°, pass through this point. Numerical solutions with higher microfibril angles at the neck show a more complicated relationship between neck radius and fiber curvature (Fig S2B). The neck radius decreases with increasing fiber curvature (see e.g. the set of solutions for μ = 65°) and even starts oscillating for solutions close to the cylinder (black dashed lines). The relationship between neck radius and the fiber (or sphere) radius of curvature of these surfaces can be solved exactly for a given L and R (Table S1). For double spheres the curvature is a function

of neck radius, g_0, (Fig S3 B-D). For surfaces constructed with 3 or more sphere segments (Fig S3 E-G) the surfaces can be parameterized by l_s, which is the height of the largest sphere segment. The black line in Fig S2, corresponding to μ_0 = 0°, is the limit case which can be described by a segment of a torus. Combining the standard equation of a torus with the boundary conditions at $z = L/2$ gives a relation between neck radius and curvature as

$$g_0 = R \pm \sqrt{\frac{1}{{\kappa_T}^2} - \frac{L^2}{4}} \pm \frac{1}{|\kappa_T|}. \qquad \textbf{[S-9]}$$

The limit values of curvature corresponding to the end points of the black line (Fig S2) are given by the dashed lines ${g_0}^{lim}$ = 1 ± $2R/L$ and ${\kappa_T}^{lim} = \pm 2R/L$.

3) Comparison with Delaunay Surfaces

The profiles calculated using equations **11a** and **11b**, are compared to Delaunay surfaces calculated using the equations of Gillette and Dyson [1] and plotted in Fig. S4 for different neck radii. The profiles are surprisingly similar. Differences become only apparent when the mean curvature of the surfaces are compared. This indicates that fiber supported surfaces can closely approximate Delaunay constant mean curvature surfaces, however with different signs of internal pressure as discussed in the main text.

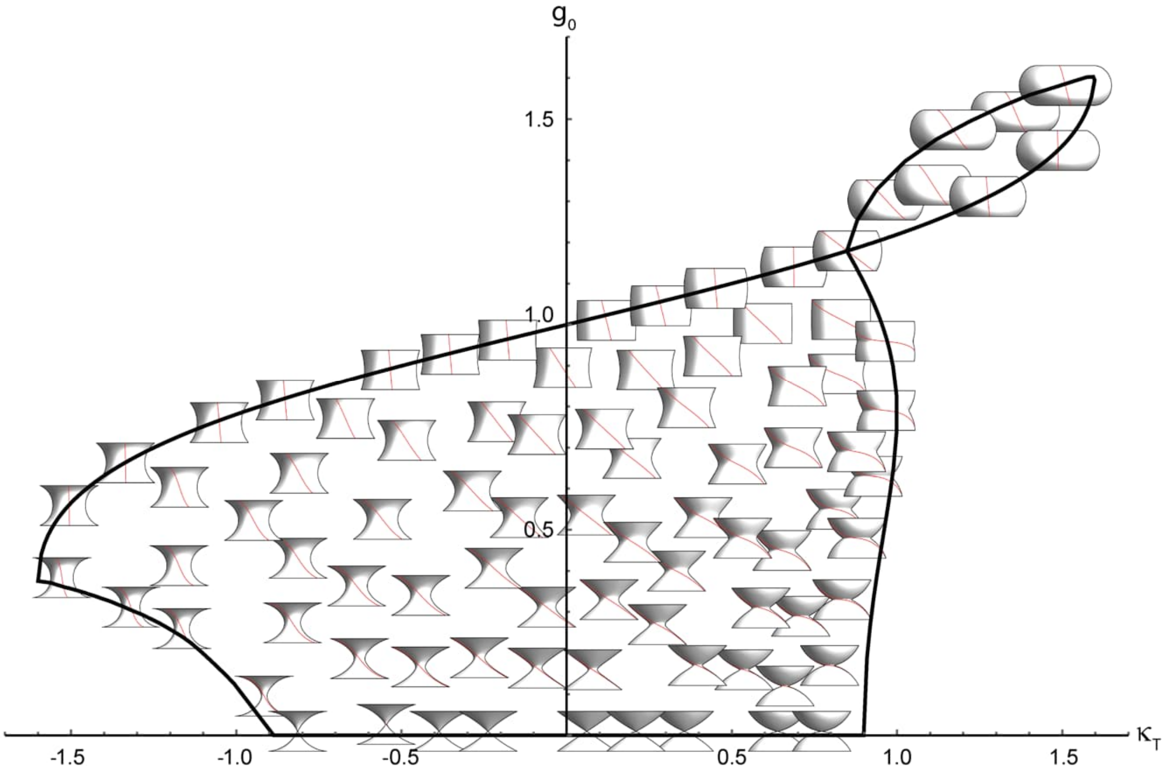

Figure S1 – Images of a selection of surfaces of revolution and their constant curvature fiber generators (red), satisfying equations **11a** and **11b** are plotted as a function of their neck radii and fiber curvature (L = 1.25, R = 1). The outer boundary (**blacklines**) describe the boundaries of the solution space in Fig **2** in the Main Manuscript.

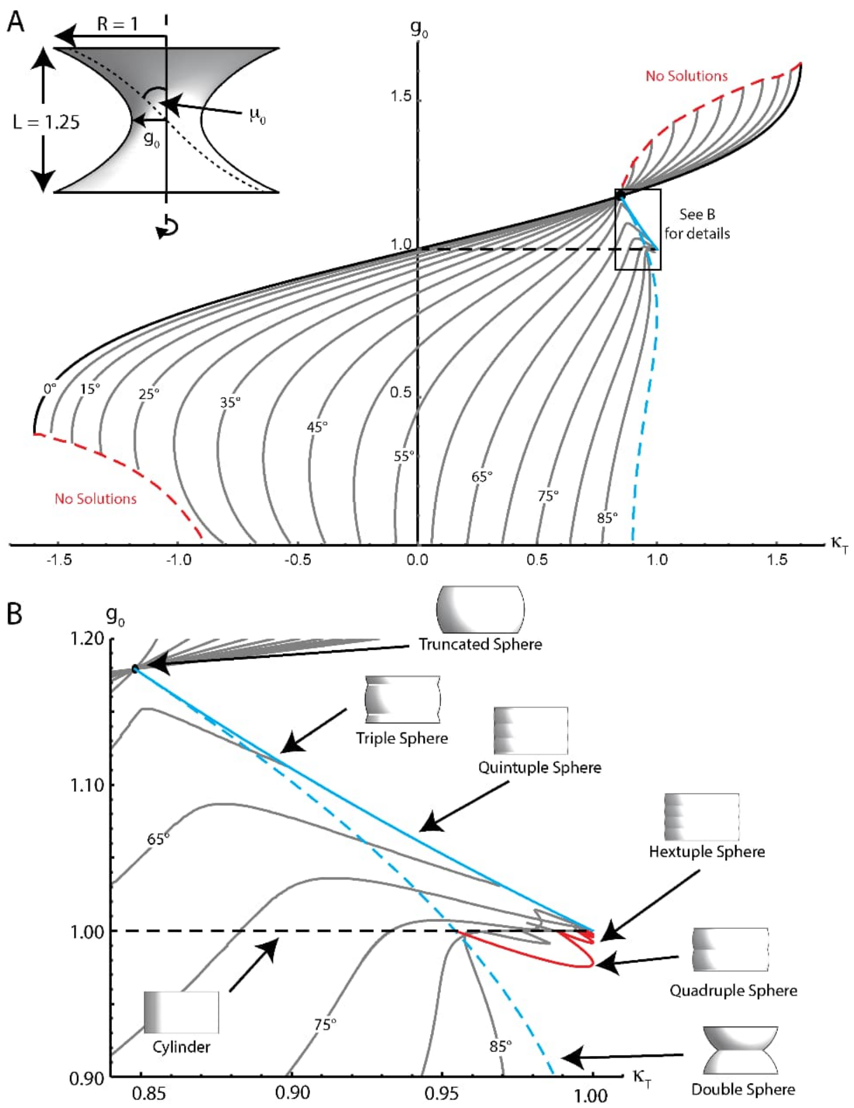

Figure S2 – Numerical solutions of neck-radius g0, versus fiber curvature κ_T A) The normalized neck radius versus the fiber curvature for surfaces of revolution satisfying equation(s) **11a** and **11b** (L =1.25, R = 1). Grey lines give the numerical solutions in which the microfibril angle at the neck is fixed at the angle indicated. The dashed red lines indicate the limits below or above which no solutions can be found. The dashed black line shows the range of cylindrical solutions. The black circle is the truncated sphere solution that satisfies equations **11a** and **11b**. The dotted blue line indicates the double truncated sphere solutions. The solid blue line indicates the parameters of triple truncated sphere solutions. The inset shows an exemplary solution B) A zoom of the boxed region from part A), shows in addition solutions for quadruple and hextuple spheres (Red) and a detail of the numerical solutions for microfibrils of 60° to 85°. Note surfaces made of an odd number of sphere segments (triple and quintuple sphere surfaces) have parameters lying on the same solid blue line.

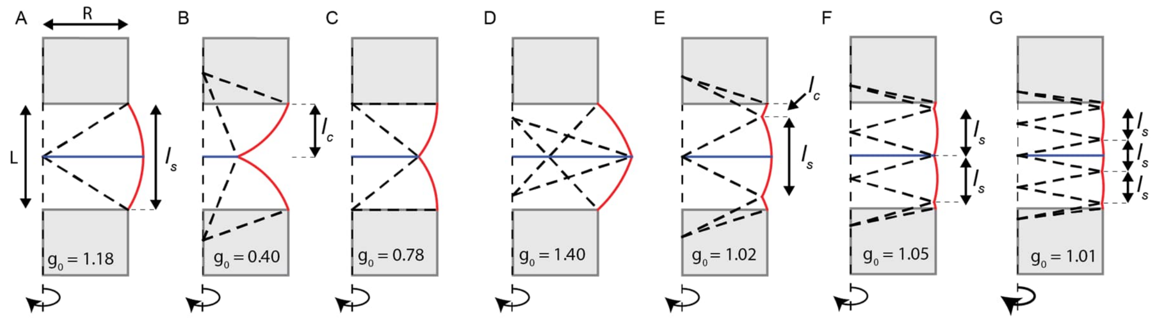

Fig. S3 – Profiles of stacked spherical segment solutions according to equations **11a** and **b**, for different neck radii. A) Truncated sphere, B), C), and D) Double sphere for different neck radii, E) Triple sphere, F) Quadruple sphere, and G) Quintuple sphere. Red lines indicate the profile curve, blue lines give the neck radius (g_0), and the dashed lines indicate the direction to the center of curvature of the different segments of the profiles. For surfaces constructed with 3 or more segments (E-G), the height of the largest sphere segment, l_s, is highlighted, l_c is the height of the smallest sphere segment.

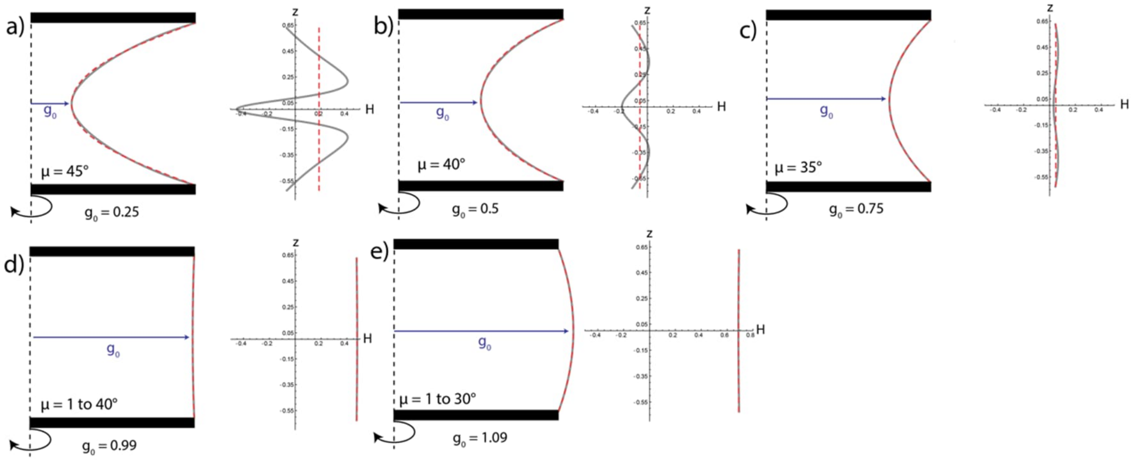

Fig. S4 – a-e (left) Profiles for different neck radii of numerical solutions according to Equations **11a** and **11b** in gray, compared to Delaunay surfaces in red. For each profile the mean curvature as a function of z is also plotted for both types of surface.

n	l_s	l_c	g_0	$(R_T = 1/\kappa_T)$
0	L	0	$\sqrt{R^2 + \frac{1}{4}L^2}$	g_0
1	0	$\frac{L}{2}$	$0 \leq g_0$	$\sqrt{R^2 + \frac{1}{L^2}(R^2 - g_0{}^2 + L^2)^2}$
2	$\frac{L}{2} \leq l_s \leq L$	$(L - l_s)/2$	$\sqrt{R^2 + \frac{1}{4}(2l_s - L)^2}$	g_0
3	$\frac{L}{3} \leq l_s \leq \frac{L}{2}$	$(L - l_s)/2$	$\sqrt{R^2 + \frac{1}{4}(3l_s - L)^2}$	$\sqrt{R^2 + \frac{1}{4}(3l_s - L)^2 - \left(\frac{l_s}{2}\right)^2}$
Even	$\frac{L}{n+1} \leq l_s \leq \frac{L}{n-1}$	$(L - l_s)/2$	$\sqrt{R^2 + \frac{1}{4}(nl_s - L)^2}$	g_0
Odd	$\frac{L}{n+1} \leq l_s \leq \frac{L}{n-1}$	$(L - l_s)/2$	$\sqrt{R^2 + \frac{1}{4}(nl_s - L)^2}$	$\sqrt{R^2 + \frac{1}{4}(nl_s - L)^2 - \left(\frac{l_s}{2}\right)^2}$

Table S1 – Relationship between the number n of stacked spheres, neck radius g_0 and fiber curvature $R_T = 1/\kappa_T$ for different stack sphere heights.

SI References

1. R. D. Gillette, D. C. Dyson, Stability of fluid interfaces of revolution between equal solid circular plates. *Chem. Eng. J.* 2, 44-54 (1971).
